# Alfred: interactive multi-sample BAM alignment statistics, feature counting and feature annotation for long- and short-read sequencing

**DOI:** 10.1093/bioinformatics/bty1007

**Published:** 2018-12-06

**Authors:** Tobias Rausch, Markus Hsi-Yang Fritz, Jan O Korbel, Vladimir Benes

**Affiliations:** bty1007-aff1 Genomics Core Facility, European Molecular Biology Laboratory (EMBL), Meyerhofstrasse 1, Heidelberg, Germany; bty1007-aff2 Genome Biology Unit, European Molecular Biology Laboratory (EMBL), Meyerhofstrasse 1, Heidelberg, Germany

## Abstract

**Summary:**

Harmonizing quality control (QC) of large-scale second and third-generation sequencing datasets is key for enabling downstream computational and biological analyses. We present Alfred, an efficient and versatile command-line application that computes multi-sample QC metrics in a read-group aware manner, across a wide variety of sequencing assays and technologies. In addition to standard QC metrics such as GC bias, base composition, insert size and sequencing coverage distributions it supports haplotype-aware and allele-specific feature counting and feature annotation. The versatility of Alfred allows for easy pipeline integration in high-throughput settings, including DNA sequencing facilities and large-scale research initiatives, enabling continuous monitoring of sequence data quality and characteristics across samples. Alfred supports haplo-tagging of BAM/CRAM files to conduct haplotype-resolved analyses in conjunction with a variety of next-generation sequencing based assays. Alfred’s companion web application enables interactive exploration of results and comparison to public datasets.

**Availability and implementation:**

Alfred is open-source and freely available at https://tobiasrausch.com/alfred/.

**Supplementary information:**

Supplementary data are available at *Bioinformatics* online.

## 1 Introduction

Many methods have been developed to perform quality control (QC) on specific types of sequencing assays (Endrullat *et al.*, 2016), such as RNA-SeQC (DeLuca *et al.*, 2012) for RNA-Seq data, Chance (Diaz *et al.*, 2012) for ChIP-seq data or Poretools (Loman and Quinlan, 2014) for Oxford Nanopore sequencing data. Popular general purpose alignment QC methods are, for instance, QualiMap2 (Okonechnikov *et al.*, 2016) and NGS QC Toolkit (Patel and Jain, 2012). Despite these developments, it remains challenging for DNA sequencing facilities and large genomics projects to find a versatile QC method that is computationally efficient, easy to use and install, robust to diverse sequencing assays as well as protocols and accessible to Bioinformaticians, experimentalists and wet-lab technicians alike. An installation-free, easy to use, interactive interface that allows exploration of the QC data across samples and data types is often not available and a direct comparison to public data frequently entails downloading gigabytes of alignment files to compute background distributions and to estimate acceptable ranges for each quality parameter.

Alfred fills an important gap in this context by computing key quality statistics rapidly, across hundreds of samples and with a versatile web front end for data exploration and comparison to publicly available data resources. For each sample, the method generates an extensible JSON file with the key QC metrics that can be merged across samples and then explored interactively at https://gear.embl.de/alfred. The Alfred command-line interface is available as a statically linked binary on GitHub, via Bioconda or as a minimal Docker container. Alfred provides a wide range of QC metrics, some of general relevance such as the insert size distribution and others more targeted to specific sequencing assays, such as the on-target rate for capture assays (Supplementary Table S1). Grouping of charts can be pursued to facilitate comparison of relevant distributions: the insert size distribution, for instance, is stratified by paired-end orientation to distinguish paired-end and mate-pair libraries frequently employed in structural variant calling (Rausch *et al.*, 2012). Alfred additionally provides fast methods for feature counting and feature annotation, generation of browser tracks and methods to analyze alignments in a haplotype-resolved manner. All of these functions are competitive with respect to runtime and memory usage compared to commonly used tools in each of these application areas (Supplementary Table S2). As input, Alfred supports BAM and CRAM files (Hsi-Yang Fritz *et al.*, 2011).

## 2 Materials and methods

Alfred uses sub-commands for BAM/CRAM statistics, feature counting and feature annotation. The main methods are outlined below. All backend code is open-source and written in C++ using HTSlib (Li *et al.*, 2009) and Boost (Schaeling, 2011).

### 2.1 BAM QC metrics

Alfred parses the BAM file only once and pre-allocates data structures for counting primary, secondary, supplementary and spliced alignments. Paired-end orientations are counted by type (F-, F+, R-, R+) and sequencing error rates are computed separately for mismatch, insertion and deletion errors (Supplementary Fig. S1). InDel sizes are cataloged and potential homopolymer sequence regions and a fragment-based GC bias curve is estimated from the reference context. If a BED file of target regions is provided, Alfred computes the on-target rate and the target coverage distribution as well as overall enrichment of targeted regions. For tagged BAM files that utilize unique molecular identifiers (UMIs), the number of UMIs and the fraction of tagged molecules are computed. For haplotype-tagged files, the number of phased blocks and the N50 phased block length are computed. All QC output files are gzip-compressed to save space. Two output formats are available: first, a block structured tab-delimited file format as in samtools stats to efficiently filter (‘grep’) desired statistics in pipelines and computational workflows and second, a succinct and extensible JSON format that can be visualized in our companion web application.

### 2.2 Feature counting and feature annotation

Alfred supports counting reads in overlapping or non-overlapping windows, at pre-defined intervals in BED format or as gene and transcript counting for RNA-Seq in stranded or unstranded mode using a gtf or gff3 gene annotation file. Expression values can be normalized as raw counts, FPKM or FPKM-UQ values. Additionally, browser tracks in UCSC bedgraph format can be computed with configurable resolution. Alfred also supports annotation of ChIP-Seq and ATAC-Seq peaks for neighboring genes or transcription factor binding sites based on motif alignments.

### 2.3 Haplotype tagging and allele-specific applications

With the advent of long reads and haplotype resolved sequencing protocols such as 10X Genomics or Strand-Seq (Porubsky *et al.*, 2017), there is an increasing need to split BAM files by haplotype and perform haplotype-aware downstream analyses. Alfred provides basic functions to haplo-tag BAM files based on phased VCF files and generates allele-specific count tables for subsequent analyses. For error-prone long read datasets, haplotype-resolved BAM files in conjunction with Alfred’s alignment methods can also be used to generate highly accurate haplotype-specific consensus sequences (Supplementary Material).

### 2.4 Multi-sample web application

Alfred’s JSON files can be visualized with the companion web application that is built with standard web technologies (HTML, CSS, JavaScript and SVG) and thus can readily be used with common web browsers. Importantly, this allows using the application from different operating systems and without any installation procedure. All charts are interactive, supporting panning and zooming and all charts and tables can be downloaded as png and csv files, respectively. Due to its client-only design (i.e. no server is involved), the application can also be installed easily and used offline or embedded in other websites, for example paper companion sites, to provide QC statistics transparently across all samples analyzed in a given study. The application is adaptive to different sequencing protocols, and several features are geared towards specific applications such as the on-target rate measurement available for capture-based sequence assays. The web application also hosts a set of JSON QC files that span a wide range of sequencing assays (DNA-Seq, RNA-Seq, ATAC-Seq, ChIP-Seq, HiC) and sequencing technologies (PacBio, Oxford Nanopore Technologies, Illumina), providing an ideal resource for researchers in need of comparing QC statistics.

## 3 Discussion

Alfred is a comprehensive alignment QC, feature counting and feature annotation method that complements specialized QC packages available for a specific sequencing assay by providing an easy to use, cross-platform interface that allows read-group aware multi-sample comparisons. Alfred supports third generation sequencing technologies and can handle 10X Genomics datasets, Strand-Seq data and reads derived from nanopore sequencing where it readily enables haplotype-resolved analyses.

## Supplementary Material

bty1007_Supplementary_Data
