# Supplementary material for: Alfred: interactive multi-sample BAM alignment statistics, feature counting and feature annotation for long- and short-read sequencing
Source: Bioinformatics. 2018 Dec 6;35(14):2489–91. doi: 10.1093/bioinformatics/bty1007 (PMC6612896; doi:10.1093/bioinformatics/bty1007)
Supplement: bty1007_Supplementary_Data [file bty1007_supplementary_data.pdf]

Supplementary Information
Alfred: Interactive multi-sample BAM alignment statistics, feature counting and feature annotation for long- and short-read sequencing

BAM statistics, feature counting and feature annotation

Alfred provides a wide range of QC metrics, some of general relevance such as the insert size distribution and others more targeted to specific sequencing assays, such as the on-target rate for capture assays (Figure 1). A list of alignment statistics provided for each sequencing assay is available in Table 1. As input, Alfred accepts BAM and CRAM files [Hsi-Yang Fritz *et al.*, 2011]. Alfred's functions are competitive with respect to runtime and memory usage compared to commonly used tools in each of its application areas (Table 2). As exemplary applications, we

1. calculated quality control statistics for a whole-exome sample with multiple read-groups,
2. computed read-depth at a given set of target intervals using DNA sequencing data,
3. performed gene counting for an RNA sequencing data set, and
4. generated down-sampled browser tracks for a ChIP sequencing data set.

For each application, we compared Alfred v0.1.12 to a commonly used method in that category, specifically QualiMap v2.2.1 [Okonechnikov *et al.*, 2016], Bedtools v2.27.1 [Quinlan & Hall, 2010], htseq-count v0.11 [Anders *et al.*, 2015] and Homer v4.9.1 [Heinz *et al.*, 2010].

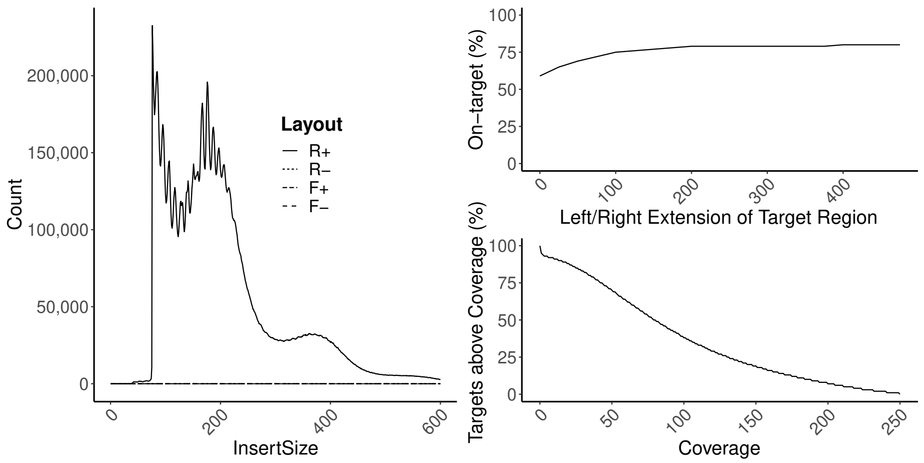

Figure 1: Exemplary Alfred Charts: Nucleosome pattern and DNA pitch for an ATAC-Seq library (left) and on-target rate as well as target coverage distribution for a whole-exome capture library (right).

Alignment Metric	DNA-Seq WGS	DNA-Seq WES/Capture	RNA-Seq	ChIP-Seq or ATAC-Seq	Chart Type
Mapping Statistics	✓	✓	✓	✓	Table
Duplicate Statistics	✓	✓	✓	✓	Table
Sequencing Error Rates	✓	✓	✓	✓	Table
Base Content Distribution	✓	✓	✓	✓	Grouped Line Chart*
Read Length Distribution	✓	✓	✓	✓	Line Chart*
Base Quality Distribution	✓	✓	✓	✓	Line Chart*
Coverage Histogram	✓	✓	✓	✓	Line Chart*
Insert Size Distribution	✓	✓	✓	✓	Grouped Line Chart*
InDel Size Distribution	✓	✓	✓	✓	Grouped Line Chart*
InDel Context	✓	✓	✓	✓	Bar Chart*
GC Content	✓	✓	✓	✓	Grouped Line Chart*
On-Target Rate		✓			Line Chart*
Target Coverage Distribution		✓			Line Chart*
TSS Enrichment				✓	Table
DNA pitch / Nucleosome pattern				✓	Grouped Line Chart*
Spliced Alignments			✓		Line Chart*
Feature Counting	✓	✓	✓	✓	Table
Feature Annotation	✓	✓	✓	✓	Table

*Interactive charts

Table 1: Alignment Statistics

Application	Alfred	QualiMap2	Bedtools	htseq-count	Homer
DNA WES QC	412s (0.8GB)	916s (3.3GB)			
DNA Loci Read Depth	131s (0.8GB)		533s (0.1GB)		
RNA Feature Counting	95s (0.2GB)	1252s (1.4GB)		2055s (1.6GB)	
ChIP-Seq Browser Tracks	616s (1.2GB)				636s (0.5GB)

Table 2: Runtime (in seconds) and peak memory usage (in gigabytes) for selected applications

Haplotype-resolved consensus computation

Alfred implements functionality to analyze sequence alignments in a haplotype-resolved manner. As an example, we illustrate in Figure 2 a dotplot of the haplotype-specific consensus sequences derived from error-prone long reads of a heterozygous complex structural variant previously identified in Phase 3 of the 1000 Genomes Project [Sudmant *et al.*, 2015]. We first split the long read data set into haplotype-specific BAM files using Alfred's `split` subcommand. This function takes a BAM file and a phased SNP backbone as input and then outputs two haplotype-specific BAM files. For each haplotype-specific BAM file, we then used Alfred's consensus function to collect all reads at a given alignment position to compute a haplotype-specific consensus sequence [Rausch *et al.*, 2009]. The consensus computation has three processing steps: (1) Building of a multiple sequence alignment guide tree using pairwise overlap alignments with affine gap penalties [Gotoh, 1986], (2) progressive alignment of all sequences along the guide tree, and (3) derivation of a consensus sequence from this multiple sequence alignment. Notably, the average sequencing error rate was 10.6% and 10.9% for the individual long reads in each of the two haplotypes. The consensus computation greatly improves this error rate to 3.5% and 3.8%. The two haplotype-specific consensus sequences can then be aligned using Alfred's pairwise alignment algorithm (`pwalign` subcommand). The `pwalign` command generates a linear alignment using dynamic programming, where the relative order of nucleotides in each sequence is preserved. Such an alignment is ideal for insertions and deletions but fails to capture more complex rearrangements such as inversions. To visualize complex rearrangements a classical dotplot can be used, and the dotplot of the haplotype-specific consensus sequences is shown in Figure 2.

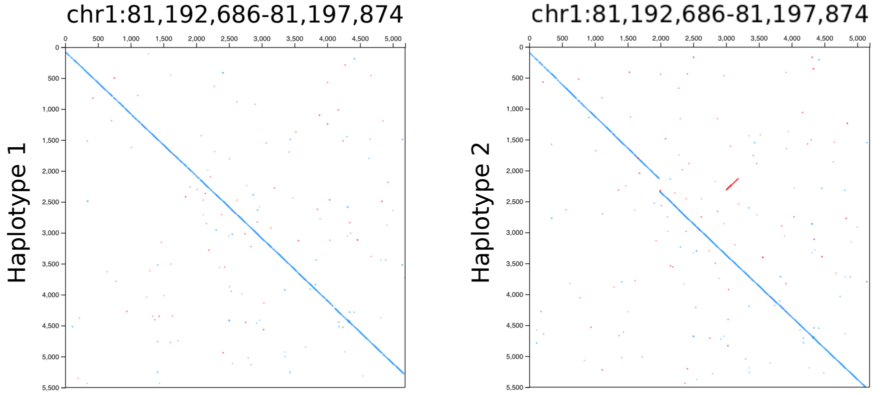

Figure 2: Haplotype-specific dotplots: dotplot alignments of haplotype1 consensus sequence compared to the reference (left) and haplotype2 consensus sequence compared to the reference (right). In haplotype2 an inverted duplication occurred.

References

[Anders *et al.*, 2015] Anders, S., Pyl, P. T. & Huber, W. (2015) HTSeq–a Python framework to work with high-throughput sequencing data. *Bioinformatics,* **31** (2), 166–169.

[Gotoh, 1986] Gotoh, O. (1986) Alignment of three biological sequences with an efficient traceback procedure. *J. Theor. Biol.,* **121** (3), 327–337.

[Heinz *et al.*, 2010] Heinz, S., Benner, C., Spann, N., Bertolino, E., Lin, Y. C., Laslo, P., Cheng, J. X., Murre, C., Singh, H. & Glass, C. K. (2010) Simple combinations of lineage-determining transcription factors prime cis-regulatory elements required for macrophage and B cell identities. *Mol. Cell,* **38** (4), 576–589.

[Hsi-Yang Fritz *et al.*, 2011] Hsi-Yang Fritz, M., Leinonen, R., Cochrane, G. & Birney, E. (2011) Efficient storage of high throughput DNA sequencing data using reference-based compression. *Genome Res.,* **21** (5), 734–740.

[Okonechnikov *et al.*, 2016] Okonechnikov, K., Conesa, A. & Garcia-Alcalde, F. (2016) Qualimap 2: advanced multi-sample quality control for high-throughput sequencing data. *Bioinformatics,* **32** (2), 292–294.

[Quinlan & Hall, 2010] Quinlan, A. R. & Hall, I. M. (2010) BEDTools: a flexible suite of utilities for comparing genomic features. *Bioinformatics,* **26** (6), 841–842.

[Rausch *et al.*, 2009] Rausch, T., Koren, S., Denisov, G., Weese, D., Emde, A. K., Doring, A. & Reinert, K. (2009) A consistency-based consensus algorithm for de novo and reference-guided sequence assembly of short reads. *Bioinformatics,* **25** (9), 1118–1124.

[Sudmant *et al.*, 2015] Sudmant, P. H., Rausch, T., Gardner, E. J., Handsaker, R. E., Abyzov, A., Huddleston, J., Zhang, Y., Ye, K., Jun, G., Fritz, M. H., Konkel, M. K., Malhotra, A., Stutz, A. M., Shi, X., Casale, F. P., Chen, J., Hormozdiari, F., Dayama, G., Chen, K., Malig, M., Chaisson, M. J. P., Walter, K., Meiers, S., Kashin, S., Garrison, E., Auton, A., Lam, H. Y. K., Mu, X. J., Alkan, C., Antaki, D., Bae, T., Cerveira, E., Chines, P., Chong, Z., Clarke, L., Dal, E., Ding, L., Emery, S., Fan, X., Gujral, M., Kahveci, F., Kidd, J. M., Kong, Y., Lameijer, E. W., McCarthy, S., Flicek, P., Gibbs, R. A., Marth, G., Mason, C. E., Menelaou, A., Muzny, D. M., Nelson, B. J., Noor, A., Parrish, N. F., Pendleton, M., Quitadamo, A., Raeder, B., Schadt, E. E., Romanovitch, M., Schlattl, A., Sebra, R., Shabalin, A. A., Untergasser, A., Walker, J. A., Wang, M., Yu, F., Zhang, C., Zhang, J., Zheng-Bradley, X., Zhou, W., Zichner, T., Sebat, J., Batzer, M. A., McCarroll, S. A., Mills, R. E., Gerstein, M. B., Bashir, A., Stegle, O., Devine, S. E., Lee, C., Eichler, E. E. & Korbel, J. O. (2015) An integrated map of structural variation in 2,504 human genomes. *Nature,* **526** (7571), 75–81.
